# Unraveling the interplay between daily life fatigue and physical activity after subarachnoid hemorrhage: an ecological momentary assessment and accelerometry study

**DOI:** 10.1186/s12984-023-01241-5

**Published:** 2023-09-26

**Authors:** Elisabeth A. de Vries, Majanka H. Heijenbrok-Kal, Fop van Kooten, Marco Giurgiu, Gerard M. Ribbers, Rita J.G. van den Berg-Emons, Johannes B.J. Bussmann

**Affiliations:** 1 Erasmus MC, Department of Rehabilitation Medicine, University Medical Center Rotterdam, Rotterdam, the Netherlands; 2 Rijndam Rehabilitation, Rotterdam, the Netherlands; 3 Erasmus MC, Department of Neurology, University Medical Center Rotterdam, Rotterdam, the Netherlands; 4 Institute for Sports and Sports Science, Mental mHealth lab, Karlsruhe Institute of Technology, Karlsruhe, Germany

**Keywords:** Fatigue, Physical activity, Subarachnoid hemorrhage, Ecological momentary assessment, Accelerometry, Rehabilitation, Stroke

## Abstract

**Background:**

Fatigue is one of the most commonly reported symptoms after subarachnoid hemorrhage (SAH) and is indirectly associated with physical activity (PA). Associations between fatigue and PA are primarily examined based on conventional measures (i.e. a single fatigue score or average PA levels), thereby assuming that fatigue and PA do not fluctuate over time. However, levels of fatigue and PA may not be stable and may interrelate dynamically in daily life. Insight in direct relationships between fatigue and PA in daily life, could add to the development of personalized rehabilitation strategies. Therefore we aimed to examine bidirectional relationships between momentary fatigue and PA in people with SAH.

**Methods:**

People (n = 38) with SAH who suffer from chronic fatigue were included in an observational study using Ecological Momentary Assessment (EMA) and accelerometry. Momentary fatigue was assessed on a scale from 1 to 7 (no to extreme fatigue), assessed with 10–11 prompts per day for 7 consecutive days using EMA with a mobile phone. PA was continuously measured during this 7-day period with a thigh-worn Activ8 accelerometer and expressed as total minutes of standing, walking, running and cycling in a period of 45 min before and after a momentary fatigue prompt. Multilevel mixed model analyses including random effects were conducted.

**Results:**

Mean age was 53.2 years (SD = 13.4), 58% female, and mean time post SAH onset was 9.5 months (SD = 2.1). Multilevel analyses with only time effects to predict fatigue and PA revealed that fatigue significantly (p < 0.001) increased over the day and PA significantly (p < 0.001) decreased. In addition, more PA was significantly associated with higher subsequent fatigue (β = 0.004, p < 0.05) and higher fatigue was significantly associated with less subsequent PA (β=-0.736, p < 0.05). Moreover, these associations significantly differed between participants (p < 0.001).

**Conclusions:**

By combining EMA measures of fatigue with accelerometer-based PA we found that fatigue and PA are bidirectionally associated. In addition, these associations differ among participants. Given these different bidirectional associations, rehabilitation aimed at reducing fatigue should comprise personalized strategies to improve both fatigue and PA simultaneously, for example by combining exercise therapy with cognitive behavioral and/or energy management therapy.

**Supplementary Information:**

The online version contains supplementary material available at 10.1186/s12984-023-01241-5.

## Background

About 30–90% of people with subarachnoid hemorrhage (SAH) suffer from chronic fatigue, [1–4] which may interfere with participation in daily life activities and health-related quality of life [5–7]. To date, there is no effective rehabilitation program specifically aimed at fatigue in patients with SAH or stroke [8]. Recent studies have shown that people with aneurysmal subarachnoid hemorrhage (A-SAH) have lower levels of physical activity (PA) than healthy controls [9] and that PA is indirectly associated with fatigue in this group. Those who are less physically active, have worse physical fitness, [10] which is associated with higher levels of fatigue [11]. The relationship between PA and fatigue also has been established in studies on mild traumatic brain injury [12] and other types of stroke [13, 14]; people who are more physically active experience less fatigue in the acute and chronic phase after injury, but findings on longitudinal association between PA and fatigue after stroke are inconclusive [13, 15–18].

Associations between fatigue and PA after stroke are primarily assessed in studies with a cross-sectional design [14]. Consequently, these studies do not provide insight in causal relationships. Furthermore, it is often assumed that both fatigue and PA are constant or trait constructs that do not fluctuate over time with fatigue expressed in a single score based on retrospective questions or PA represented as an average level derived from questionnaires or accelerometer data [12–18]. However, levels of fatigue and PA may not be stable between and within days and they may interrelate dynamically in daily life [19–22]. By simultaneously assessing fatigue and PA multiple times per day, using for example Ecological Momentary Assessment (EMA) and accelerometry, direct relationships between fatigue and PA in daily life might be revealed that would have been hidden when measured otherwise [20, 22].

In a study that combined EMA data on fatigue (assessed 10 times a day) and diary-based PA scores, people with stroke reported higher fatigue levels when they reported more PA before the fatigue prompt and when they perceived activities as more effortful. In addition to this overall effect, interindividual differences were found in the increase in fatigue with more effortful daily activities [23]. In patients with Multiple Sclerosis (MS) bidirectional relationships were found between momentary fatigue (assessed 5 times a day) and accelerometer-based PA; higher fatigue was followed by less PA and more PA was associated with a subsequent decline in fatigue. However, this latter relationship was not constant over the day; in the morning and evening more PA was followed by a subsequent decline in fatigue, but during midday more PA was followed by a subsequent increase in fatigue [24]. The differences between the studies in people with stroke and MS may be related to the different diagnosis groups, but also to differences in the study methods (e.g. different EMA prompting schemes and diary-based vs. accelerometer-based PA assessment).

Insight in real-life momentary relationships between fatigue and PA in people with SAH is lacking, but could add to the development of personalized rehabilitation programs ultimately increasing the effectiveness of interventions aimed at fatigue. For example, if people with SAH instantly reduce their PA in response to fatigue, rehabilitation aimed at coping with fatigue might help them to continue their activities despite their fatigue, or for people who experience more fatigue in the afternoon, rehabilitation in the morning might be more effective. Therefore, the aim of this study was to examine bidirectional relationships between momentary fatigue and device based assessed PA in people with SAH. We hypothesized that more PA is followed by higher subsequent fatigue and that higher fatigue predicts less subsequent PA.

## Methods

### Participants and procedures

Consecutive patients with SAH, who were treated at Erasmus MC University Medical Centre Rotterdam (Augustus 2017-May 2019) and patients treated at Elisabeth-TweeSteden Hospital (January 2019-January 2020) were screened for eligibility. Inclusion criteria were: at least 18 years of age, 3 to 12 months post SAH onset, living at home and experiencing fatigue since SAH (checked by EV in a telephone call). Exclusion criteria were: previous stroke or suffering from another severe chronic and/or neurological disease, other clinical reason for fatigue, insufficient mastery of the Dutch language and inability to fill in the electronic diary. After inclusion a researcher visited the participants at home, where a semi-structured interview was conducted to assess baseline characteristics (e.g. smoking status, years of education) and participants received instructions for filling in the electronic diary and wearing the accelerometer. Participants wore the accelerometer for 7 consecutive days and they filled in the electronic diary during the same days. For descriptive purposes, before the home-visit participants were asked to fill in the fatigue severity scale (FSS) questionnaire. All participants provided written informed consent before inclusion. The medical ethics committee of Erasmus MC (MEC-2017-523) approved the study.

### Momentary fatigue

Momentary fatigue was assessed with an electronic diary using the MovisensXS application on a mobile phone (Alcatel U3), that participants received the day before the measurement week. Patients were asked 10–11 times per day “How fatigued do you feel at this moment?” (scale ranging from 1 (not fatigued) to 7 (extremely fatigued), which corresponds with the scale of the FSS questionnaire [25]. Participants were prompted at random time points between 09:00 and 21:00. Based on previous studies [23, 26, 27], it is reasonable to assume that fatigue will not fluctuate considerably in a period of less than 45 min. Therefore, we chose 45 min as minimum time interval between two prompts.

### Physical activity

PA was assessed with the Activ8 accelerometer (2M Engineering B.V. Valkenswaard, the Netherlands), which is a small (30 × 32 × 10 mm) and light-weighted (20 g) device that was attached on the front of the upper thigh with Tegaderm™ skin tape. The Activ8 is validated [28] and found to be accurate in detecting body postures and movements in patients with stroke [29]. Participants were asked to daily fill out the time they went in and out of bed in a paper diary, which were used to define waking time. Also, they were asked to write down any comments when a day was not representative of usual activities (e.g. lying sick in bed the whole day) and to note periods of non-wear. Participants were allowed to bath and swim while wearing the Activ8.

The Activ8 has a tri-axial accelerometer which detects 6 postures or movements: lying, sitting, standing, walking, cycling, running. PA was defined as the combined category of standing, walking, running and cycling. Data was continuously collected for 24 h/day during 7 consecutive days and summarized and stored in 30 s epochs. In this study only waking time was considered. Total time in minutes of PA in a period of 45 min prior and after a fatigue prompt was calculated. A time interval of 45 min was chosen since this was the minimum time period between two fatigue prompts and in this way we were able to make maximum use of the Activ8 data.

### Descriptive measures

For descriptive purposes, trait fatigue was assessed with the validated and widely used Fatigue Severity Scale (FSS) [25, 30]. The FSS consists of nine statements about the impact of fatigue in daily life during the previous week. Statements are scored on a 7-point Likert Scale, ranging from 1 (strongly disagree) to 7 (strongly agree), where the total score is the mean of these 9 items. Fatigued patients were distinguished from non-fatigued patients using a cut-off score of at least 4 [25]. Clinical characteristics (e.g. type of SAH, duration of hospital stay, smoking history) were collected from the patient files and semi-structured interviews.

### Statistical analysis

RStudio Version 1.4.1103 was used for all statistical analyses. Descriptive statistics were used to check statistical assumptions. Baseline characteristics of the participants and descriptive statistics of fatigue and PA were described by mean values and standard deviations for variables on an interval scale or frequencies and percentages for categorical variables. If participants showed less than 30% EMA compliance, data were not valid based on EMA guidelines and excluded from the analyses. In addition, irrespective of number of waking hours, if Activ8 data was not representative based on the paper diary of the participants, that day was excluded from the analyses. Periods of non-wear as indicated by the participants in the paper diary were also excluded.

We conducted multilevel-mixed models with repeated measurements of fatigue and PA (level-1) nested within participants (level-2). First, we examined the course of fatigue and PA over the day by conducting two models with either fatigue or PA as dependent variable and time and time^2^ as covariates. Time^2^ effects were included to take into account possible non-linear effects of time on fatigue and PA over the day, if that improved model fit. In addition, we examined whether random slopes for time significantly contributed to the model fit. Subsequently, we examined univariable associations between baseline characteristics and fatigue and PA, by conducting separate models with fatigue or PA as dependent variable and the given characteristic and time effects as covariates.

Finally, to examine bidirectional associations between fatigue and PA, two multivariable models were built: one model with fatigue as dependent variable and PA time in the 45 min period *before* the fatigue prompt as covariate and one model with PA time in the 45 min period *after* the fatigue prompt as dependent variable and momentary fatigue score as covariate. The momentary covariates fatigue and PA were person-mean centered, thereby modelling the momentary change from a person’s usual fatigue / PA level. Other covariates of interest in both models were, time, time^2^ and day type (week day or weekend day). The models were adjusted for sex, age and the baseline characteristics that were significant (p < 0.05) in the univariable models. For each of the models we examined whether interactions of either fatigue or PA with time significantly contributed to the model fit based on the Bayesian Information Criteria. Also, we examined whether interactions of fatigue or PA with the characteristic(s) that were significantly associated with fatigue or PA in the univariable models significantly contributed to the model fit. We added random intercepts and slopes for time and either PA time or fatigue and a continuous time auto-regressive correlation structure to the model, to account for the correlations in the data. Model assumptions, (i.e. approximately 95% of standardized residuals between − 1.96 and 1.96 and normally distributed residuals and random effects), were met. The two final models are presented in Additional file 1.

## Results

### Participants

In total, 42 patients participated in the study. One patient did not reach minimal EMA compliance of 30% and was therefore excluded from the analyses. In addition, Activ8 data of 3 participants were missing, one Activ8 was damaged and two were lost, resulting in a total group of 38 participants for the analyses, with a mean age of 53.2 years (SD = 13.4) and 9.5 months (SD = 2.1) post SAH onset. Mean FSS score of the group was 5.11 (SD = 1.18) and 31 patients (82%) were fatigued based on the cut-off score of 4. Baseline characteristics of all participants are presented in Table 1.

### Momentary fatigue

**Fig. 1:**
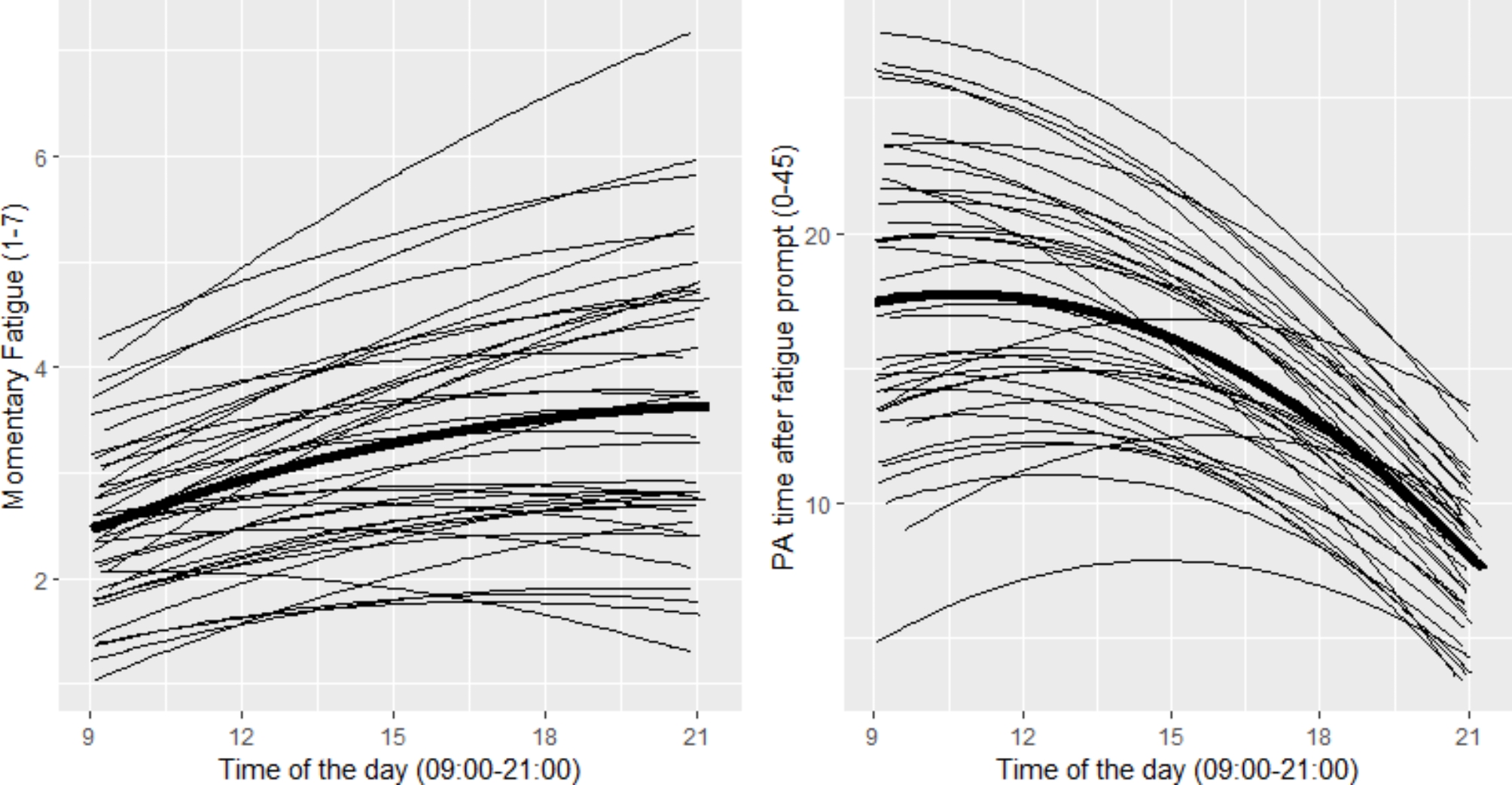
Course of momentary fatigue (left panel) and physical activity in 45 min after the fatigue prompt (right) over the day separately for all participants and for the whole group (bold line)

Mean momentary fatigue of the total group was 3.22 (SD = 1.46). Mean momentary fatigue at the first prompt, at 09:36 AM (SD = 21 min), was 2.51 (SD = 1.16) and at the last prompt, at 08:40 PM (SD = 17 min), was 3.76 (SD = 1.55). The left panel of Fig. 1 shows the regression lines of fatigue over the day for all participants separately and for the whole group, demonstrating that fatigue significantly increased over the day (time; β = 0.30, p < 0.001) and leveled off at the end of the day (time^2^; β=-0.007, p < 0.001) and that this course significantly differed among participants (random slopes for time; p < 0.001). In univariable analyses we found that smokers had significantly higher momentary fatigue than non-smokers (β = 0.72, p < 0.05) and that momentary fatigue was significantly higher on week days than in the weekend (β=-0.18, p < 0.01). For all other baseline characteristics no significant associations with momentary fatigue were found.

**Table 1:** Baseline characteristics

	Total Group (N = 38)
**Sociodemographic characteristics**	
Age at onset, years, mean (SD)	53.2 (13.4)
Time since SAH onset, months, mean (SD)	9.5 (2.1)
Female, n (%)	22 (57.9)
Living with others, n (%)	34 (89.5)
Education, years, mean (SD)	14.2 (5.5)
History of smoking, yes, n (%)	17 (44.7)
Currently smoking, yes, n (%)	9 (23.7)*
**Clinical characteristics**	
Type SAH, n (%)	
Aneurysmatic	30 (78.9)
Perimesencephalic	8 (21.1)
Duration of hospital stay, days, mean (SD)	13.4 (6.3)†
Hypertension, yes, n (%)	6 (15.8)
WFNS grade, n (%)	
Good (1–2)	33 (86.8)†
Poor (3–5)	5 (13.2)
Location of aneurysm, n (%)	
Anterior circulation	21 (77.7)
Posterior circulation	6 (22.2)
Treatment modality aneurysm, n (%)	
Endovascular	23 (85.2)†
Neurosurgical	4 (14.8)
Serious SAH complications, yes, n (%)	11 (29.0)
Discharge destination, n (%)	
Home	30 (78.9)
Inpatient clinic	8 (21.1)

Abbreviations: SAH, Subarachnoid Hemorrhage; WFNS, World Federation of Neurosurgical Societies. Notes: Three participants of the aneurysmatic group suffered a non-aneurysmal SAH. Serious SAH complications; none vs. hydrocephalus or ischemia. Inpatient clinic comprised rehabilitation center or nursing home. *Significant association with momentary fatigue, †Significant association with physical activity

### Physical activity

Of all 266 measurement days, 5 days (1.9%) were considered as non-representative based on the notes of the participants in the paper diary. Mean PA of the total group per day was almost 5 h (297 min, SD = 89), which is 32.6% of the mean total analyzed time per day of 15.2 h (SD = 1.0) (i.e. awake time minus periods of non-wear which was 4.0 min, SD = 7.8). Mean PA time in the 45 min *before* the fatigue prompt was 15.41 (SD = 13.3) minutes (34.2%) and in the 45 min *after* the fatigue prompt 14.96 (SD = 13.0) minutes (33.2%). In the right panel of Fig. 1 the regression lines of PA time in the 45 min *after* the fatigue prompts over the day for all participants separately and for the whole group (bold line) are presented. Mean PA time in the 45 min *after* the first fatigue prompt, at 09:36 AM (SD = 21 min), was 18.16 (SD = 13.1). Mean PA time in the 45 min *after* the last fatigue prompt, at 8:40 PM (SD = 17 min), was 8.08 (SD = 9.1) minutes. PA time in the 45 min after the fatigue prompt slightly increased at the start of the day (time; β = 1.99, p < 0.001) and subsequently decreased over the day (time^2^; β=-0.09, p < 0.001) and this course significantly differed among participants (random slopes for time; p < 0.001). Univariable analyses showed that longer stay at the hospital (β=-0.24), poor WFNS score (good, β = 4.70) and neurosurgical treatment (β=-4.74) were significantly (p < 0.05) associated with less PA. For all other baseline characteristics no significant associations with PA were found.

### Physical activity and subsequent fatigue

The details of the multivariable model with fatigue as outcome variable are presented in Table 2. More than usual PA in the 45 min prior to a fatigue prompt was significantly (p < 0.05) associated with higher subsequent fatigue (β = 0.004), adjusted for time, time^2^, age, sex, type of day, duration of hospital stay, WFNS score and smoking status. In addition, the change in fatigue after more PA differed among participants (random slopes for PA; p < 0.05). Fatigue was significantly higher on week days than on weekend days (β=-0.223, p < 0.001). The other covariates in the model (i.e. age, sex, duration of hospital stay, WFNS category and smoking status) were not independently associated with fatigue. The interaction effects of PA with the time variables and with day type and smoking status did not significantly improve the model fit.

### Fatigue and subsequent physical activity

The details of the multivariable model with PA as outcome variable are presented in Table 2. Higher than usual fatigue was significantly associated with less PA (β=-0.736) in the 45 min after the fatigue prompt (p < 0.05), adjusted for time, time^2^, age, sex, type of day, duration of hospital stay, WFNS score and smoking status. In addition, the change in PA after higher fatigue levels differed among participants (random slopes for fatigue; p < 0.05). Both poor WFNS score (good; β = 4.589, p < 0.05) and being a smoker (β=-4.001, p < 0.01) were associated with lower PA. The other covariates in the model (i.e. age, sex and duration of hospital stay) were not independently associated with PA. The interaction effects of fatigue with the time variables and with duration of hospital stay and WFNS category did not significantly improve the model fit.

## Discussion

By combining prospective EMA measures of fatigue and accelerometer-based PA in daily life, we found direct relationships between fatigue and PA in people with SAH, thereby adding new insights to existing literature on cross-sectional associations between trait fatigue and mean PA levels [12, 13, 18]. As hypothesized, more than usual PA was directly associated with higher subsequent momentary fatigue and people with SAH exhibited less PA instantly following higher than usual fatigue levels. Moreover, the interplay between fatigue and PA differed among participants.

When we compare our findings to EMA literature on the relationships between fatigue and PA, similarities and differences can be noticed. For example, our findings on the positive relationship between PA and subsequent fatigue are in line with results from an EMA study in people with stroke that showed that more PA was followed by higher fatigue [23]. In people with MS this relationship was also found, however only during midday; in the morning and evening after being physically active a reduction in fatigue was found [24]. A negative relationship between PA and fatigue was also found in healthy adults, where spending more time in light PA relative to no PA resulted in lower subsequent fatigue [31]. Regarding the relationship between fatigue and subsequent PA, previous EMA studies in people with MS [24] and healthy people [31] showed that people who experience higher than usual fatigue reduce their subsequent PA, which is consistent with our findings. It has to be noted, however, that previous studies used different designs (e.g. operationalization of PA, different definitions of PA and different EMA prompting schemes), which might influence the results and conclusions and may hinder direct comparisons.

Contrary to what has been found in healthy people [31], we found that more PA was directly associated with higher fatigue in people with SAH. This might be explained by differences in physical fitness levels of the individuals [32, 33].People with SAH are found to have lower physical fitness levels than healthy controls [34], which was found to be associated with trait fatigue and PA [10, 11, 34]. Consequently, PA in daily life might be more effortful for people with SAH than for healthy people, resulting in higher fatigue directly after being more physically active [35]. Exercise training to improve physical fitness, might reduce fatigue or prevent high post-activity fatigue, especially since people with SAH are found to be less physically active than healthy controls and often seems not to adhere to the PA and exercise recommendations for stroke survivors [9, 36]. Also, this may aid in breaking the vicious circle of deconditioning, that proposes that fatigue results in physical inactivity, leading to lower physical fitness and even more fatigue [33, 35]. In future studies, it would be interesting to examine the effect of personalized exercise training on this potential mechanism and on momentary relationships between fatigue and PA.

Besides physiological mechanisms as described in the previous paragraph, psychological mechanisms may also play a role in the interplay between fatigue and PA in daily life. People with SAH might avoid PA due to fear of worsening their fatigue or recurrence of SAH or because they believe that fatigue diminishes after resting [37, 38], which might explain the finding that higher fatigue was associated with less subsequent PA. This is consistent with findings from qualitative studies where fatigue was the most common barrier for being physically active after stroke [39, 40]. In addition, the level of self-efficacy might also play a role in the bidirectional association between fatigue and PA; a previous EMA study in healthy adults showed that higher self-efficacy predicts more subsequent PA [41], while in people with SAH [42] and stroke [14], lower self-efficacy is associated with higher fatigue and less PA.

The bidirectional interplay between fatigue and PA in daily life found in this study highlights the importance of developing rehabilitation strategies simultaneously targeting fatigue and PA to eventually reduce fatigue. Behavioral interventions addressing aspects as energy management and activity pacing strategies seem important to balance PA and fatigue levels. In addition, cognitive interventions targeting coping with fatigue, self-efficacy and kinesiophobia (e.g. by providing information about SAH and the chances of recurrence of SAH) seem useful to prevent avoidance behavior and increase the likelihood of performing PA despite experiencing fatigue [38]. Such a multidisciplinary approach aligns with findings that interventions targeting one aspect such as exercise therapy, cognitive behavioral therapy or energy management often yield inconclusive outcomes on fatigue in people with neurological conditions [8, 43–50], while multidisciplinary interventions have demonstrated promising results on reducing fatigue in people with stroke [51] and chronic fatigue syndrome [52]. Recently, ecological momentary interventions (EMIs) in combination with EMA of symptoms receive attention in improving strategies to enhance healthy behavior [53] and mental health [54]. In order to affect behavior or feelings in daily life, in response to a given momentary state such as a period of inactivity or high fatigue level (EMA), both active prompts (e.g. stand up and walk for one minute) and passive prompts (e.g. information or tips on coping with fatigue) can be provided on a persons’ mobile phone (EMI) [53, 54]. In future studies it would be interesting to examine whether EMIs can add to current interventions targeting fatigue and PA simultaneously after SAH.

This study has some limitations that need to be considered. First of all, PA before and after a fatigue prompt of people with SAH in our study was rather low. In future studies, it would be interesting to study the relationship between fatigue and PA using activity-based EMA prompts. Participants are then prompted after a given amount of minutes of PA or no PA. This will increase the insight in the effect of longer bouts of PA on fatigue. In addition, the sample size of our study was relatively small, however, given the many repeated measurements within persons, this sample size was considered appropriate for modelling associations between PA and fatigue [55]. In addition, we included only people with SAH who suffer from fatigue in this study. Still, overall momentary fatigue of the participants was rather low in comparison to their trait fatigue. Therefore, the results may be less generalizable for people experiencing higher momentary fatigue in daily life. Finally, previous studies showed that the context of being physically active in daily life, such as with or without company or gardening instead of cycling on a home trainer, might affect symptom experience [20]. In future studies it would be interesting to assess this context, to provide even more specific targets for out-patient rehabilitation programs.

## Conclusions

In conclusion, by combining EMA measures of fatigue with accelerometer-based PA we found that momentary fatigue and PA are bidirectionally associated in people with SAH. People with SAH reduced their PA in response to fatigue and more than usual PA resulted in higher levels of fatigue. In addition, the relationships between fatigue and PA differed among participants. Given the dynamic interplay between fatigue and PA in daily life and the differences between participants in this interplay, these findings should be taken into account in developing personalized rehabilitation programs aimed at reducing fatigue and enhancing PA after subarachnoid hemorrhage.

**Table 2:** Bidirectional associations between fatigue and physical activity

	Model 1: Fatigue as dependent variable				Model 2: PA time as dependent variable			
Fixed effects	β (SE)	95% CI	t-value (df)	p-value	β (SE)	95% CI	t-value (df)	p-value
Intercept	0.507 (0.993)	-1.441–2.454	0.510 (2557)	0.610	2.827 (6.355)	-9.634–15.288	0.445 (2557)	0.656
Time	**0.291 (0.058)**	**0.177–0.406**	**4.995 (2557)**	**< 0.001**	**2.077 (0.700)**	**0.703–3.450**	**2.965 (2557)**	**0.003**
Time^2^	**-0.006 (0.002)**	**-0.010–-0.003**	**-3.402 (2557)**	**< 0.001**	**-0.093 (0.023)**	**-0.137–-0.048**	**-4.074 (2557)**	**< 0.001**
Age, Years	-0.001 (0.011)	-0.023–0.024	0.093 (32)	0.926	-0.010 (0.043)	-0.077–0.097	0.240 (32)	0.812
Sex, Female	0.011 (0.308)	-0.616–0.638	0.037 (32)	0.971	1.693 (1.144)	-0.638–4.024	1.479 (32)	0.149
Day type, Weekend	**-0.223 (0.062)**	**-0.345–-0.101**	**-3.591 (2557)**	**< 0.001**	-0.148 (0.598)	-1.321–1.025	-0.247 (2557)	0.805
Hospital stay, Days	-0.020 (0.026)	-0.072–0.032	-0.771 (32)	0.446	-0.135 (0.094)	-0.327–0.057	-1.432 (32)	0.162
WFNS, Good	-0.101 (0.454)	-1.025–0.824	-0.222 (32)	0.826	**4.589 (1.711)**	**1.104–8.075**	**2.682 (32)**	**0.012**
Smoking, Yes	0.696 (0.367)	-0.051–1.443	1.897 (32)	0.067	**-4.001 (1.363)**	**-6.778–-1.225**	**-2.936 (32)**	**0.006**
PA time	**0.004 (0.002)**	**0.003–0.015**	**2.416 (2557)**	**0.016**	-	-	-	-
Fatigue	-	-	-	-	**-0.736 (0.306)**	**-1.335–-0.137**	**-2.408 (2557)**	**0.016**

Notes: Time in hours, range 9:00 h-21:00 h. PA time in minutes, range 0–45. Bold indicates significant (p<0.05) associations. Abbreviations: WFNS, World Federation of Neurosurgical Societies; PA, physical activity; SE, Standard Error; 95% CI, 95% Confidence Interval; df, degrees of freedom

### Electronic supplementary material

Below is the link to the electronic supplementary material.

**Additional file** Additional file 1: Final models analysis (DOCX)

## Data Availability

The datasets generated and/or analysed during the current study are available from the corresponding author on reasonable request.

## List of abbreviations

SAH: Subarachnoid Hemorrhage
PA: Physical Activity
EMA: Ecological Momentary Assessment
MS: Multiple Sclerosis
FSS: Fatigue Severity Scale
WFNS: World Federation of Neurosurgical Societies
