# Supplementary material for: Unraveling the interplay between daily life fatigue and physical activity after subarachnoid hemorrhage: an ecological momentary assessment and accelerometry study
Source: J Neuroeng Rehabil. 2023 Sep 26;20:127. doi: 10.1186/s12984-023-01241-5 (PMC10521384; doi:10.1186/s12984-023-01241-5)
Supplement: Supplementary file 1 — Additional file Additional file 1: Final models analysis (DOCX) [file 12984_2023_1241_MOESM1_ESM.docx]

**Additional file 1: Final models analysis**

Model 1: ${Fatigue}_{ij}= \beta_{0}+ \beta_{1}{Time}_{ij}+ \beta_{2}{Time}_{ij}^{2}+ \beta_{3}{Sex}_{i}+ {\beta_{4}Age}_{i}+ {\beta_{5}Day Type}_{i}+ {\beta_{6}PAtime}_{i}+ {\beta_{7}WFNS}_{i}+ {\beta_{8}HospitalStay}_{i} + {\beta_{9}Smoking}_{i}+ b_{io}+b_{i1}{Time}_{ij}+b_{i1}{PAtime}_{ij}+ɛ_{ij}$

Model 2: ${PAtime}_{ij}= \beta_{0}+ \beta_{1}{Time}_{ij}+ \beta_{2}{Time}_{ij}^{2}+ \beta_{3}{Sex}_{i}+ {\beta_{4}Age}_{i}+ {\beta_{5}Day Type}_{i}+ {\beta_{6}Fatigue}_{i}+{\beta_{7}WFNS}_{i}+ {\beta_{8}HospitalStay}_{i} + {\beta_{9}Smoking}_{i}+ b_{io}+b_{i1}{Time}_{ij}+b_{i1}{Fatigue}_{ij}+ɛ_{ij}$
